# Associated factors with umbilical arterial pH after cesarean delivery under spinal anesthesia: a retrospective cohort study

**DOI:** 10.1016/j.bjane.2021.04.022

**Published:** 2021-04-28

**Authors:** Miwa Kitaguchi, Mitsuru Ida, Yusuke Naito, Yuka Akasaki, Masahiko Kawaguchi

**Affiliations:** Nara Medical University, Department of Anesthesiology, Kashihara, Japan

**Keywords:** Anesthesia, spinal, Arterial pressure, mean, Cesarean section, Ephedrine, Fetal blood, Hydrogen-ion concentration

## Abstract

**Background:**

Maximum decrease of blood pressure and number of minutes of hypotension were independently associated with umbilical arterial pH. However, the impact of hypotension considering the duration of it on umbilical arterial pH is unknown.

**Methods:**

Pregnant women aged ≥ 20 years who delivered a baby at full-term via a cesarean delivery under a single-shot spinal anesthesia between January 2017 and March 2019 were included. The main outcome was to predict umbilical arterial pH, based on the value of the time integral of hypotension. Patient demographics, patient comorbidities, and intraoperative data, including the total dose of ephedrine and phenylephrine by fetal delivery and cumulative duration of maternal hypotension, were evaluated. Maternal hypotension was reflected as a decrease in systolic arterial pressure and mean arterial pressure to < 80% of baseline values. The systolic arterial pressure and mean arterial pressure were independently included in a multiple regression analysis along with all other explanatory factors to predict the umbilical arterial pH.

**Results:**

Of the 416 eligible patients, 381 were enrolled. When including the systolic arterial pressure or mean arterial pressure in the model, emergency cases, the total dose of ephedrine, hypertensive disorders of pregnancy, and systolic arterial pressure or mean arterial pressure values were found to be significant predictive factors of umbilical arterial pH.

**Conclusion:**

Our results suggest that an elevated time integral of maternal hypotension may have a negative impact on umbilical arterial pH. Therefore, to minimize the risk of fetal acidosis, maternal hypotension should be prevented with the consideration of vasopressors selection.

## Introduction

Hypotension induced by spinal anesthesia for a cesarean delivery is very common in women in both healthy and complicated pregnancies, which causes adverse effects such as nausea, vomiting and syncope.^1^ Maternal hypotension also decreases placental blood flow, leading to the deterioration in fetal well-being, as indicated by Apgar scores and umbilical arterial pH (UA pH).^2^, ^3^, ^4^ Therefore, it is a medical imperative to prevent and efficiently treat hypotension following spinal anesthesia. In addition to fluid administration and left lateral uterine displacement, the use of α-agonist drugs is recommended in current clinical practice.^5^

Some systematic reviews and meta-analyses have assessed the effects of vasopressors on UA pH^6^, ^7^, ^8^; however, few studies have focused on the duration and severity of maternal hypotension during a cesarean delivery performed under spinal anesthesia.^9^, ^10^ One study published in 1982 concluded that hypotension lasting under 2 minutes did not affect newborn outcomes, but the number of patients was limited (n = 31) and none of them experienced hypotension lasting longer than 2 minutes.^9^ Another study published in 2003 reported that a maximum decrease in systolic arterial pressure (SAP) and a duration of hypotension defined as SAP value < 80% of its baseline value were independently associated with UA pH.^2^ However, in both studies were conducted a long time ago and the impact of hypotension considering both magnitude and duration on UA pH was not evaluated.

There is no unique, well-accepted definition of hypotension in obstetric anesthesia. In a literature search performed from 1999 to 2009, 15 different definitions of hypotension were found spanning 63 articles.^10^ An international consensus statement published in 2018 stated that SAP should be maintained at ≥ 90% of its baseline value obtained before spinal anaesthesia.^5^ Additionally, the importance of mean arterial pressure (MAP) over SAP is stressed; however, it remains unknown which blood pressure variables and threshold characteristics are most related to fetal outcomes.^5^, ^11^

Therefore, we performed a retrospective analysis to evaluate the impact of maternal hypotension, considering both magnitude and duration, on UA pH in pregnant women who had undergone a cesarean delivery under spinal anesthesia.

## Methods

This retrospective observational study was approved by the Nara Medical University Institutional Review Board, Kashihara, Nara, Japan (Chairperson Prof. M Yoshizumi, Approval No. 2205 on 10 October 2019) and carried out in compliance with the Declaration of Helsinki. The requirement for informed consent was waived due to the retrospective nature of this study. We opted out to ensure that pregnant women had the opportunity to refuse the study.

Pregnant women aged ≥ 20 years who delivered babies born at-term via a cesarean delivery performed with a single shot spinal anesthesia at Nara Medical University between January 2017 and March 2019 were eligible for the study. Multiple pregnancies, cases which were converted to general anesthesia, cases requiring multiple administrations of spinal anesthesia, and cases receiving epidural anesthesia were excluded. Pregnant women who received oxygen administration other than via a face mask or nasal cannula, and cases which had missing maternal and infant data were also excluded from the analysis.

Our institution protocol for cesarean delivery is as follows. After attaching standard anesthesia monitors, spinal anesthesia was administered with 2–2.5 mL of hyperbaric bupivacaine (0.5%), 10 μg of fentanyl and 100 μg of morphine with the patient in a lateral position. Maternal blood pressure was oscillometrically (YP-963T, NIHON KOHDEN, Shinjuku, Tokyo, Japan) measured prior to anesthetic induction and at 1-minute intervals after induction until fetal delivery. Intraoperative blood pressure management was carried out at the discretion of each anesthesiologist.

**Figure 1:**
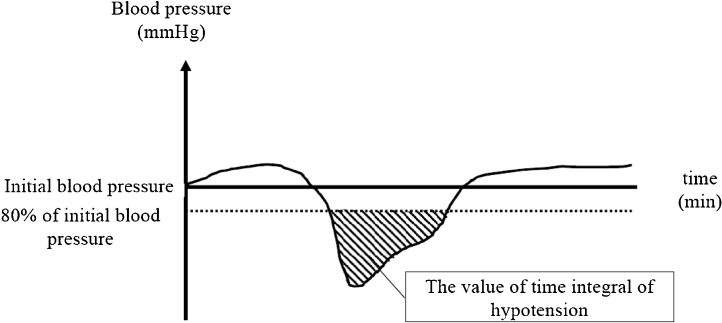
The value of the time integral of hypotension.

We assessed maternal demographic data, including age, body mass index, gestational weeks, smoking status during pregnancy (none, passive smoking, current smoking), presence of a hypertensive disorder of pregnancy, presence of diabetes mellitus (none, diabetes mellitus before pregnancy, and gestational diabetes mellitus), and thyroid function during pregnancy (normal, hyperthyroidism, and hypothyroidism). Additionally, having undergone elective or emergency surgery, having indication for a cesarean delivery, the total dose of ephedrine and phenylephrine administered by fetal delivery, oxygen administration by fetal delivery, the value of the hypotension time integral (Fig. 1), umbilical arterial pH, neonatal Apgar scores at 1 and 5 minutes postdelivery, and neonatal weight were retrieved from anesthetic records and electronic medical records. As the number of pregnant women with comorbidities and experiencing an emergency cesarean delivery is increasing, we included those patients in this study in order to reflect daily clinical practice.

This study aimed to explore associated factors with UA pH focused on the time integral value of maternal hypotension. And maternal hypotension was defined as a decrease in SAP or mean arterial pressure (MAP) reaching levels < 80% of the initial values measured in the operating room. UA pH was measured from a blood sample taken after the umbilical cord was doubly clamped at the time of delivery using a Stat Profile® pHOx® Ultra device (Nova Biomedical, Minato, Tokyo, Japan)

### Statistical analysis

Data are presented as mean (standard deviation) or numbers (percentages). A multiple regression analysis was used to evaluate associated factors with UA pH, in which all explanatory factors except for indications for cesarean delivery were included along with the time integral value of SAP or MAP to adjust maternal comorbidity and surgical situation. Additionally, as sensitivity analyses, multiple regression models were carried out which included SAP or MAP values reaching < 90% or < 100% of their initial values, respectively. All data were analyzed using SPSS version 22.0 (IBM Inc., Armonk, NY, USA), and *p*-values < 0.05 were considered indicative of statistical significance.

Considering 15 covariates included in the multiple regression analysis, the required sample size, calculated using G*power version 3.1 (Faul, Erdfelder, Lang, & Buchner, 2007) with the requirements of a type I (α) error, power (1-β) and effect size (f^2^) of 0.05, 0.95, and 0.15 (medium effect size), respectively, was found to be 199 patients. Estimating the attrition rate to be 30% and considering the number of surgeries performed at our hospital, we decided to review 27 months of patient data.

## Results

**Figure 2:**
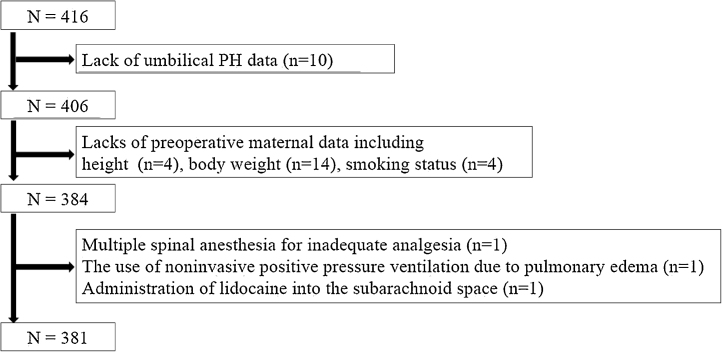
Flowchart.

During the study period, of 416 eligible pregnancies, complete data was available for 381 patients, all of which were included in the analysis (Fig. 2). As a result, we were able to include more patients than was required for the estimated sample size, which means that confidence intervals were calculated more accurately.

As shown in Table 1, 37.5% underwent an emergency surgery and the average UA pH was 7.28. Table 2 shows the indications for cesarean delivery. Table 3, Table 4 show the results of the multiple regression analysis, including SAP or MAP values < 80% of their initial values. In both models, having undergone an emergency surgery, presence of a hypertensive disorder of pregnancy, increased dose of ephedrine, and a large hypotension time integral were found to be associated factors of UA pH. The results of the sensitivity analyses also revealed similar results (Supplementary Tables 1–4).

**Table 1:** Descriptive summary of variables.

	Mean ± standard deviation or number (%) (n = 381)
Age (y)	33.8 ± 4.8
Body Mass Index (kg.m^-2^)	26.7 ± 5.1
Gestational day (days)	266 ± 8.8
Hypertensive disorder of pregnancy	27 (7.1)
Smoking status during pregnancy	
None	292 (76.7)
Passive smoking	65 (17.1)
Current smoking	24 (6.2)
Diabetes mellitus	
None	331 (86.8)
Diabetes mellitus before pregnancy	14 (3.7)
Gestational diabetes	36 (9.4)
Thyroid function during the pregnancy	
Normal	355 (93.1)
Hyperthyroidism	8 (2.1)
Hypothyroidism	18 (4.7)
Emergency surgery	143 (37.5)
Dose of ephedrine until delivery (mg)	2.5 ± 4.1
Dose of phenylephrine until delivery (mg)	0.16 ± 0.18
Oxygen administration until delivery	46 (12.1)
Umbilical arterial pH	7.28 ± 0.04
Apgar score at 1 min	8.5 ± 1.1
Apgar score at 5 min	9.6 ± 0.8
Birth weight (g)	2976 ± 458

**Table 2:** Indications for a cesarean delivery.

	Number (n = 381)
Previous cesarean section	180
Prolonged and obstructed labor	48
Hypersensitive disorder of pregnancy	8
Premature membrane rupture	6
Threatened premature birth	3
Placenta previa	11
Low-lying placenta	8
Uterine myoma during pregnancy	3
History of intrauterine procedures	17
General disease complicating pregnancy	3
Threatened uterine rupture	5
Chorioamnionitis	1
Post-dated pregnancy	3
Symphyseolysis in pregnancy	1
Malpresentation	51
Congenital anomalies	2
Cephalopelvic disproportion	6
Non-reassuring fetal status	25

**Table 3:** Results of multiple regression analysis including systolic arterial pressure reaching < 80% of its initial value.

	Regression coefficient (β)	Standard error	95% Confidence Interval (lower limit, upper limit)	*p*-value
Constant	7.432			
Age (y)	-0.00008	< 0.001	-0.001, 0.001	0.85
Body Mass Index (kg.m^-2^)	0.0004	< 0.001	0.000, 0.001	0.39
Gestational day (days)	-0.001	< 0.001	-0.001, < 0.001	0.07
Hypertensive disorder of pregnancy	-0.016	0.008	-0.031, < 0	0.045
Smoking status during pregnancy				
None	Reference			
Passive smoking	-0.001	0.006	-0.013, 0.11	0.91
Current smoking	0.013	0.009	-0.005, 0.03	0.16
Diabetes mellitus				
None	Reference			
Diabetes mellitus before pregnancy	-0.019	0.012	-0.043, 0.005	0.12
Gestational diabetes	0.009	0.01	-0.006, 0.025	0.21
Thyroid function during pregnancy				
Normal	Reference			
Hyperthyroidism	0.007	0.01	-0.024, 0.037	0.66
Hypothyroidism	-0.013	0.015	-0.033. 0.007	0.21
Emergency surgery	-0.015	0.005	-0.026, -0.004	0.006
Dose of ephedrine until delivery (mg)	-0.002	0.001	-0.03, -0.01	0.001
Dose of phenylephrine until delivery (mg)	-0.016	0.012	-0.04, 0.008	0.2
Oxygen administration until delivery	0.004	0.007	-0.01, 0.017	0.59
The value of time integral of hypotension	0.00003	< 0.001	< 0.000, < 0.000	0.047

**Table 4:** Results of multiple regression analysis including mean arterial pressure reaching < 80% of its initial value.

** **	Regression coefficient (β)	Standard error	95% Confdential Interval (lower limit, upper limit)	*p*-value
Constant	7.434			
Age (y)	-0.0001	< 0.001	-0.001, 0.001	0.78
Body Mass Index (kg.m^-2^)	0.0004	< 0.001	0.000, 0.001	0.34
Gestational day (days)	-0.001	< 0.001	-0.001, < 0.001	0.068
Hypertensive disorder of pregnancy	-0.016	0.008	-0.031, < 0	0.046
Smoking status during pregnancy				
None	Reference			
Passive smoking	-0.001	0.006	-0.013, 0.11	0.87
Current smoking	0.012	0.009	-0.006, 0.025	0.17
Diabetes mellitus				
None	Reference			
Diabetes mellitus before pregnancy	-0.019	0.012	-0.043, 0.005	0.12
Gestational diabetes mellitus	0.009	0.008	-0.006, 0.025	0.21
Thyroid function during pregnancy				
Normal	Reference			
Hyperthyroidism	0.007	0.015	-0.023, 0.037	0.64
Hypothyroidism	-0.012	0.010	-0.032. 0.008	0.24
Emergency surgery	-0.015	0.005	-0.026, -0.004	0.006
Dose of ephedrine until delivery (mg)	-0.002	0.001	-0.03, -0.01	0.001
Dose of phenylephrine until delivery (mg)	-0.015	0.012	-0.039, 0.009	0.22
Oxygen administration until delivery	0.004	0.007	-0.01, 0.017	0.58
The value of time integral of hypotension	0.00001	< 0.001	< 0.000, < 0.000	0.029

## Discussion

Our study’s results demonstrated that in pregnant women who had undergone a cesarean delivery under spinal anesthesia, a large hypotension time integral, defined according to various parameters, resulted in a decreased UA pH. Furthermore, the significant associated factors with UA pH included having undergone emergency surgery, presence of a hypertensive disorder of pregnancy, having used an increased dose of ephedrine, and a large hypotension time integral. Some studies reported the impact of hypotension UA pH had not been evaluated; however, there is no previous study reporting the impact of the time integral value of maternal hypotension.^2^, ^9^ Different definitions of hypotension have been used in the field of obstetric anesthesia.^10^ An absolute SAP value of 90 or 100 mmHg is an easy index to use without knowing a patient’s baseline blood pressure^12^; however, some pregnant women may already have SAP values prior to spinal anesthesia which are lower than these definite SAP values. In fact, in our cohort, 47 (12.3%) patients had SAP values under 100 mmHg prior to anesthetic induction. Therefore, a percentage decrease from baseline blood pressure may be a reasonable index to use. In addition, uterine blood flow is determined by the mean uterine arterial pressure, which is likely to be more affected by MAP than by SAP; however, studies focused on MAP remain sparse.^11^, ^13^ Furthermore, one retrospective analysis which included a total of 919 pregnant women who had undergone a cesarean delivery showed that nearly half of patients experienced transient hypotension, with blood pressure values reaching < 30% of their baseline values; however, there were no significant differences in Apgar scores at 1 minute, whether patients experienced maternal hypotension or not (5 vs. 4; *p* =  0.74).^14^ However, this study did not consider the duration of hypotension and, in light of high fetal oxygen consumption and the fact that placental blood vessels lack autoregulation, even a small decrease in maternal blood pressure can cause fetal acidosis.^15^ Therefore, we analyzed the impact of hypotension considering its magnitude and duration. As expected, our results demonstrated that, regardless of the definition of hypotension used, the larger the value of the time integral of hypotension, the lower the UA pH. This may imply that baseline blood pressure levels should be maintained in to avoid fetal acidosis.

Our finding demonstrating that the use of ephedrine is associated with a lower UA pH is consistent with the results of previous studies and is explained by a higher placental transfer of ephedrine, and, thereby, fetal metabolic hyperactivity.^7^, ^16^ Additionally, emergency cases and the presence of hypertensive disorders of pregnancy were independently associated with UA pH, which suggested that we need to be more careful to avoid maternal hypotension in this situation. Because an extremely urgent cesarean delivery is performed on patients under general anesthesia in our institution, our cohort did not include mother–infant pairs with placental functions that excessively reduce oxygen supply such as in the case of a placental abruption. In fact, this might be explained by the lowest value of UA pH of 7.09. However, there would be various factors related to lower UA pH in emergency cases. Furthermore, patients with hypertensive disorders of pregnancy experience less frequent and less severe hypotension during spinal anesthesia and the sympathetic blockade caused by spinal anesthesia improves blood flow by decreasing uteroplacental resistance.^17^, ^18^ However, in addition to placental dysfunction by incomplete spiral artery remodeling resulting in decreasing oxygen supply to the fetus, excessive maternal hypotension might cause reduce blood flow.^19^, ^20^

There were several limitations to this study. First, in our study, baseline blood pressure was defined as the blood pressure measured in the operating room prior to anesthetic induction. A previous study commented that carrying out repeated measurements and averaging these blood pressure values is time-consuming and difficult to adopt in routine clinical practice, especially in emergency situations.^21^ Second, given the retrospective nature of the study, other important factors influencing UA pH such as uterine incision-to-delivery time were not able to be included in the analysis.

## Conclusion

We performed a retrospective multiple regression analysis to predict UA pH in pregnant women who had undergone a cesarean delivery under spinal anesthesia. Having undergone an emergency surgery, presence of a hypertensive disorder of pregnancy, an increased dose of ephedrine, and a large time integral of hypotension were found to be significant predictors of UA pH. In addition to the SAP, the MAP was also found to be related to UA pH. Maternal blood pressure should be kept at baseline blood pressure levels and the administration of vasopressors should be considered to minimize the risk of fetal acidosis.

## Conflicts of interest

The authors declare no conflicts of interest.
