# Supplementary material for: Associated factors with umbilical arterial pH after cesarean delivery under spinal anesthesia: a retrospective cohort study
Source: Braz J Anesthesiol. 2021 Apr 28;72(4):466–71. doi: 10.1016/j.bjane.2021.04.022 (PMC9373073; doi:10.1016/j.bjane.2021.04.022)
Supplement: Supplementary file 1 [file mmc1.docx]

**BJAN-D-20-00186_Supplementary Material**

**Supplementary Table 1 -** Result of multiple regression analysis including the systolic arterial pressure to < 100% of the initial value.

|  | **Regression coefficient (β)** | **Standard error** | **95%Confdential Interval (lower limit, upper limit)** | ***p*-value** |
| --- | --- | --- | --- | --- |
| Constant | 7.443 |  |  |  |
| Age (y) | -0.00003 | < 0.001 | -0.001, 0.001 | 0.94 |
| Body Mass Index (kg.m^-2^) | 0.0005 | < 0.001 | 0.000, 0.001 | 0.27 |
| Gestational day (days) | -0.001 | < 0.001 | -0.001, < 0.001 | 0.05 |
| Hypertensive disorder of pregnancy | -0.017 | 0.008 | -0.032, < 0 | 0.034 |
| Smoking status during pregnancy | |  |  |  |
| None | Reference |  |  |  |
| Passive smoking | -0.0004 | 0.006 | -0.012, 0.11 | 0.94 |
| Current smoking | 0.011 | 0.009 | -0.006, 0.029 | 0.2 |
| Diabetes mellitus |  |  |  |  |
| None | Reference |  |  |  |
| Diabetes mellitus before pregnancy | -0.02 | 0.012 | -0.044, 0.004 | 0.1 |
| Gestational diabetes mellitus | 0.009 | 0.008 | -0.006, 0.024 | 0.23 |
| Thyroid function during pregnancy |  |  |  |  |
| Normal | Reference |  |  |  |
| Hyperthyroidism | 0.009 | 0.010 | -0.021, 0.039 | 0.56 |
| Hypothyroidism | -0.013 | 0.010 | -0.033. 0.007 | 0.21 |
| Emergency surgery | -0.015 | 0.005 | -0.026, -0.004 | 0.007 |
| Dose of ephedrine until delivery (mg) | -0.002 | 0.001 | -0.03, -0.01 | 0.001 |
| Dose of phenylephrine until delivery (mg) | -0.017 | 0.012 | -0.04, 0.007 | 0.17 |
| Oxygen administration until delivery | 0.003 | 0.007 | -0.01, 0.016 | 0.67 |
| The value of time integral of hypotension | 0.00001 | < 0.001 | < 0.000, < 0.000 | 0.031 |

**Supplementary Table 2 -** Result of multiple regression analysis including the systolic arterial pressure to < 90% of the initial value.

|  | **Regression coefficient (β)** | **Standard error** | **95%Confdential Interval (lower limit, upper limit)** | ***p*-value** |
| --- | --- | --- | --- | --- |
| Constant | 7.437 |  |  |  |
| Age (y) | -0.00002 | < 0.001 | -0.001, 0.001 | 0.96 |
| Body Mass Index (kg.m^-2^) | 0.0004 | < 0.001 | 0.000, 0.001 | 0.29 |
| Gestational day (days) | -0.001 | < 0.001 | -0.001, < 0.001 | 0.056 |
| Hypertensive disorder of pregnancy | -0.016 | 0.008 | -0.031, < 0 | 0.047 |
| Smoking status during pregnancy |  |  |  |  |
| None | Reference |  |  |  |
| Passive smoking | -0.001 | 0.006 | -0.012, 0.11 | 0.92 |
| Current smoking | 0.012 | 0.009 | -0.006, 0.03 | 0.18 |
| Diabetes mellitus |  |  |  |  |
| None | Reference |  |  |  |
| Diabetes mellitus before pregnancy | -0.019 | 0.012 | -0.043, 0.005 | 0.12 |
| Gestational diabetes mellitus | 0.009 | 0.008 | -0.006, 0.024 | 0.23 |
| Thyroid function during pregnancy |  |  |  |  |
| Normal | Reference |  |  |  |
| Hyperthyroidism | 0.008 | 0.010 | -0.022, 0.038 | 0.59 |
| Hypothyroidism | -0.013 | 0.015 | -0.033. 0.008 | 0.21 |
| Emergency surgery | -0.015 | 0.005 | -0.025, -0.004 | 0.008 |
| Dose of ephedrine until delivery (mg) | -0.002 | 0.001 | -0.03, -0.01 | 0.001 |
| Dose of phenylephrine until delivery (mg) | -0.015 | 0.012 | -0.039, 0.009 | 0.22 |
| Oxygen administration until delivery | 0.003 | 0.007 | -0.01, 0.017 | 0.65 |
| The value of time integral of hypotension | 0.00002 | < 0.001 | < 0.000, < 0.000 | 0.015 |

**Supplementary Table 3** - Result of multiple regression analysis including the mean arterial pressure to < 100% of the initial value.

|  | **Regression coefficient (β)** | **Standard error** | **95%Confdential Interval (lower limit, upper limit)** | ***p*-value** |
| --- | --- | --- | --- | --- |
| Constant | 7.443 |  |  |  |
| Age (y) | -0.00009 | < 0.001 | -0.001, 0.001 | 0.82 |
| Body Mass Index (kg.m^-2^) | 0.001 | < 0.001 | 0.000, 0.001 | 0.27 |
| Gestational day (days) | -0.001 | < 0.001 | -0.001, 0.001 | 0.054 |
| Hypertensive disorder of pregnancy | -0.0017 | 0.008 | -0.032, < 0 | 0.033 |
| Smoking status during pregnancy | |  |  |  |
| None | Reference |  |  |  |
| Passive smoking | -0.001 | 0.006 | -0.013, 0.11 | 0.91 |
| Current smoking | 0.011 | 0.009 | -0.007, 0.029 | 0.21 |
| Diabetes mellitus |  |  |  |  |
| None | Reference |  |  |  |
| Diabetes mellitus before pregnancy | -0.02 | 0.012 | -0.044, 0.004 | 0.11 |
| Gestational diabetes mellitus | 0.009 | 0.008 | -0.006, 0.024 | 0.23 |
| Thyroid function during pregnancy |  |  |  |  |
| Normal |  |  |  |  |
| Hyperthyroidism | 0.009 | 0.015 | -0.021, 0.039 | 0.24 |
| Hypothyroidism | -0.012 | 0.01 | -0.032. 0.008 | 0.56 |
| Emergency surgery | -0.015 | 0.005 | -0.026, -0.004 | 0.005 |
| Dose of ephedrine until delivery (mg) | -0.002 | 0.001 | -0.03, -0.01 | 0.001 |
| Dose of phenylephrine until delivery (mg) | -0.017 | 0.012 | -0.04, 0.007 | 0.17 |
| Oxygen administration until delivery | 0.003 | 0.007 | -0.01, 0.017 | 0.65 |
| The value of time integral of hypotension | 0.00001 | < 0.001 | < 0.000, < 0.000 | 0.025 |

**Supplementary Table 4 -** Result of multiple regression analysis including the mean arterial pressure to < 90% of the initial value.

|  | **Regression coefficient (β)** | **Standard error** | **95%Confdential Interval (lower limit, upper limit)** | ***p*-value** |
| --- | --- | --- | --- | --- |
| Constant | 7.439 |  |  |  |
| Age (y) | -0.0001 | < 0.001 | -0.001, 0.001 | 0.81 |
| Body Mass Index (kg.m^-2^) | 0.0004 | < 0.001 | 0.000, 0.001 | 0.29 |
| Gestational day (days) | -0.001 | < 0.001 | -0.001, < 0.001 | 0.059 |
| Hypertensive disorder of pregnancy | -0.016 | 0.008 | -0.031, < 0 | 0.042 |
| Smoking status during pregnancy | |  |  |  |
| None | Reference |  |  |  |
| Passive smoking | -0.001 | 0.006 | -0.013, 0.11 | 0.88 |
| Current smoking | 0.012 | 0.009 | -0.006, 0.03 | 0.20 |
| Diabetes mellitus |  |  |  |  |
| None | Reference |  |  |  |
| Diabetes mellitus before pregnancy | -0.020 | 0.012 | -0.044, 0.005 | 0.11 |
| Gestational diabetes mellitus | 0.009 | 0.008 | -0.006, 0.024 | 0.23 |
| Thyroid function during pregnancy |  |  |  |  |
| Normal | Reference |  |  |  |
| Hyperthyroidism | 0.008 | 0.015 | -0.023, 0.038 | 0.59 |
| Hypothyroidism | -0.012 | 0.010 | -0.032. 0.008 | 0.25 |
| Emergency surgery | -0.015 | 0.005 | -0.026, -0.004 | 0.006 |
| Dose of ephedrine until delivery (mg) | -0.002 | 0.001 | -0.03, -0.01 | 0.001 |
| Dose of phenylephrine until delivery (mg) | -0.015 | 0.012 | -0.039, 0.009 | 0.21 |
| Oxygen administration until delivery | 0.003 | 0.007 | -0.01, 0.017 | 0.62 |
| The value of time integral of hypotension | 0.00001 | < 0.001 | < 0.000, < 0.000 | 0.018 |
